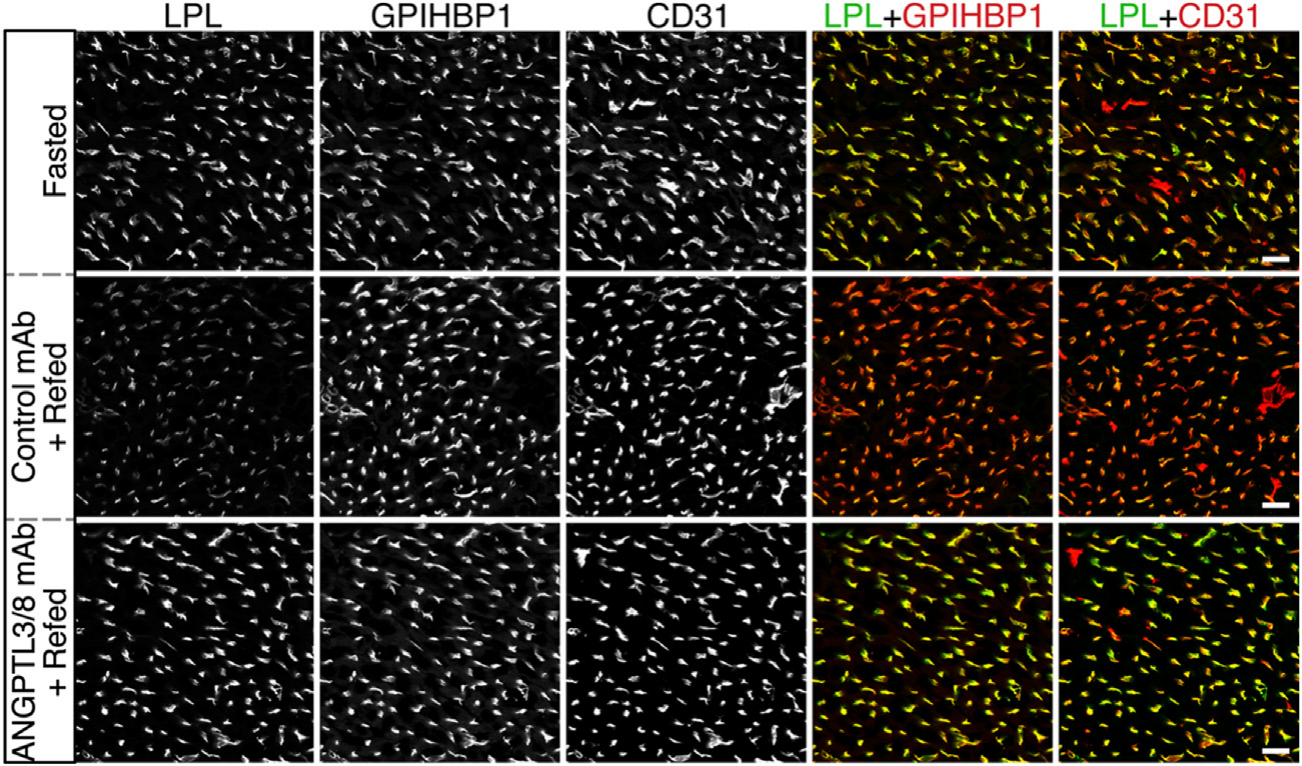# Imaging the ANGPTL3/8-mediated regulation of lipoprotein lipase in the heart

**DOI:** 10.1016/j.jlr.2023.100467

**Published:** 2023-10-28

**Authors:** Ye Yang, Hyesoo Jung, Robert J. Konrad, Loren G. Fong, Stephen G. Young

**Affiliations:** 1 Department of Medicine, University of California, Los Angeles, CA, USA; 2 Department of Human Genetics, University of California, Los Angeles, CA, USA; 3 Lilly Research Laboratories, Eli Lilly and Company, Indianapolis, IN, USA

The lipolytic processing of triglyceride (TG)-rich lipoproteins in the heart is regulated by feeding status. In 1988, Kuwajima *et al*. (1) found that the amount of TG hydrolase activity released from the heart during a heparin infusion is greater during fasting than after refeeding, but whether the observed changes in TG hydrolase activity reflected amounts of lipoprotein lipase (LPL) inside blood vessels was not assessed. Recent studies revealed that the plasma levels of the ANGPTL3/ANGPTL8 complex (ANGPTL3/8), an inhibitor of LPL catalytic activity, increase after refeeding (2). In cell culture studies, ANGPTL3/8 detaches LPL from GPIHBP1 and heparan sulfate proteoglycans (binding sites for LPL in capillaries) (3). Here, we investigated whether amounts of LPL inside heart capillaries are higher during fasting than after refeeding, and if so, whether ANGPTL3/8 is important for regulating intracapillary LPL levels after refeeding. Mice that had been fasted for 15 h were given an intravenous injection of an inhibitory ANGPTL3/8-specific monoclonal antibody (mAb IBA490) or an irrelevant control mAb (11A9) (3); the mice were then refed a chow diet for 4 h. IBA490-treated refed mice, 11A9-treated refed mice, and fasted mice were then given an intravenous injection of Alexa Fluor–labeled mAbs against LPL (27A7), GPIHBP1 (11A12), and CD31 (2H8) (3). After 10 min, the vasculature was perfused; tissues were fixed with paraformaldehyde; and sections of the left ventricular apex were imaged by fluorescence microscopy. The fluorescence intensity of 27A7, relative to that of 11A12 or 2H8, was measured in 283–1777 capillary segments/heart (n = 4 independent experiments). These studies revealed that amounts of intracapillary LPL, relative to GPIHBP1, fell by 58.2 ± 16.3% after refeeding (*P* = 0.0004). Relative to CD31, intracapillary LPL levels fell by 49.4 ± 18.4% (*P* = 0.0029). IBA490 blocked the decrease in intracapillary LPL levels after refeeding. These studies demonstrated the functional relevance of ANGPTL3/8 in regulating intracapillary LPL levels in the heart after refeeding.

**Equipment:** LSM980 confocal microscope (Zeiss).

## Conflict of interest

R. J. K. is an employee of Lilly Research Laboratories (Eli Lilly & Company) and holds stock in the company. S. G. Y. is a scientific advisory board member of Kyttaro, Inc. The other authors declare that they have no conflicts of interest with the contents of this article.

**Figure:**